# Correction: A role for descending auditory cortical projections in songbird vocal learning

**DOI:** 10.7554/eLife.04371

**Published:** 2014-08-22

**Authors:** Yael Mandelblat-Cerf, Liora Las, Natalia Denisenko, Michale S Fee

Mandelblat-Cerf Y, Las L, Denisenko N, Fee MS. 2014. A role for descending auditory cortical projections in songbird vocal learning. *eLife* **3**:e02152. doi: 10.7554/eLife.02152. Published 16 June 2014

In the published article, the author list was incorrectly given as ‘Yael Mandelblat-Cerf, Liora Las, Natalia Denissenko, Michale Fee’.

This has now been corrected.

Additionally, in Figure 1A, the two schematic diagrams were placed in the wrong order, and in Figure 1F, AIV neurons were highlighted in purple rather than in white as stated in the legend.

The image has now been corrected to switch the diagrams and highlight AIV neurons in white to match the legend.

